# Fuel Characteristics and Phytotoxicity Assay of Biochar Derived from Rose Pruning Waste

**DOI:** 10.3390/ma17081895

**Published:** 2024-04-19

**Authors:** Julia Stefanów, Karolina Sobieraj, Małgorzata Hejna, Katarzyna Pawęska, Kacper Świechowski

**Affiliations:** 1 Department of Applied Bioeconomy, Wrocław University of Environmental and Life Sciences, 37a Chełmońskiego Str., 51-630 Wrocław, Poland; 118334@student.upwr.edu.pl (J.S.); karolina.sobieraj@upwr.edu.pl (K.S.); kacper.swiechowski@upwr.edu.pl (K.Ś.); 2 Institute of Environmental Engineering, Wrocław University of Environmental and Life Sciences, 24 Grunwaldzki Square, 50-363 Wrocław, Poland; katarzyna.paweska@upwr.edu.pl

**Keywords:** biochar amendment, solid fuel, phytotoxicity, horticulture waste, pruning waste, organic waste, circular economy, organic fertilizer, pyrolysis

## Abstract

The aim of this study was the characterization and evaluation of applicability as a soil amendment of biochar derived from rose pruning waste at different pyrolysis temperatures (200–500 °C) and process durations (20–60 min). The biochar properties were compared to the raw material. The biochars produced at 300 °C for 40 and 60 min demonstrated the best fuel properties. These variants showed high energy gain rates (77.6 ± 1.5% and 74.8 ± 1.5%, respectively), energy densification ratios (1.35 ± 0.00 and 1.37 ± 0.00, respectively), high heating values (24,720 ± 267 J × g^−1^ and 25,113 ± 731 J × g^−1^, respectively), and relative low ash contents (5.9 ± 0.5% and 7.1 ± 0.3%, respectively). Regarding fertilizer properties, such as pH value, ash content, heavy metal content, and pollutant elution, the biochars showed better qualities than the raw material. All tested biochar did not exceed the permissible values for heavy metals, including Cr, Cd, Ni, and Pb. The most optimal properties for soil amendments were noted for biochar variants of 400 °C for 40 min, 450 °C for 20 min, and 500 °C for 20 min. Generally, biochars produced at temperatures ≥400 °C did not inhibit root elongation, except for the material produced at 450 °C for 60 min (4.08 ± 23.34%). Biochars obtained at ≥300 °C showed a positive impact on seed germination (86.67 ± 18.48–100 ± 24.14%).

## 1. Introduction

Due to the increasing demand for commercially cut flowers, including those produced locally, their production sector has grown in recent years [1]. In this market, with a total sale amount of almost 25 billion dollars worldwide, countries such as the Netherlands, USA, Colombia, Kenya, Zimbabwe, Japan, and Israel play the largest role [2,3]. In the European Union, the average area of horticultural farms showed an increase, reaching the highest percentage for German and Spanish crops (5.79 and 4.94% on average over 2004–2013, respectively) [4]. One of the leading European producers of pro-export nursery plants is Poland, with the acreage of floriculture increasing in the first decade of the 21st century at the rate of 7.74% annually [5].

Among the flowers that play the most important role in domestic and foreign trade, the leading position is taken by roses, with their annual world sales exceeding 730 million euros [6]. Roses are also unquestionably dominant in the Polish market since the area planted with roses in this country accounts for approximately 27% of the total area in the cut flower production sector [7]. Roses, originating from the northern hemisphere, are grown mainly in a temperate climate, but due to their numerous aesthetic and pharmaceutical applications, the cultivation of many of its species and varieties is carried out in various environments around the world [8,9]. To ensure the proper quality and yield of roses, they are subjected to a series of agrotechnical and care treatments, one of the most important of which is pruning. It not only facilitates and speeds up the collection of flowers but is also important in the more efficient removal of weeds, preventing the development of diseases and pests, as well as easier irrigation and fertilization [10].

However, the procedure of rose pruning is also related to the problem of managing the resulting waste, such as the remains of dry and semi-green stems and other plant fragments. One hectare of planted roses produces approximately 500 kg of waste annually [11]. As the crop’s area constantly continues to grow, the amount of waste biomass increases. Currently, the most common way of its disposal is accumulating in the open spaces of the production companies and burning in the field [10]. However, this method not only results in the unused potential of organic matter contained in plant residues but also contributes to the emission of pollutants, greenhouse gases, and odors into the air, as well as the leaching of potentially toxic compounds [12]. On the other hand, gardeners and crop owners do not practice introducing this green waste back into the soil directly, thus avoiding the risk of contamination with fungi and pathogen dissemination [10].

High soil organic matter content is one of the many requirements related to the production of cut roses. Any essential element deficiency can limit plant growth and lower the quantity and quality of flowers gathered [11]. However, high chemical fertilizer applications associated with the intensive production of cut flowers leads to environmental issues, such as soil salinization and water eutrophication [13]. Given the challenges associated with managing the waste from rose pruning and the fact that the successful production of these flowers depends mainly on the careful selection and application of fertilizers and microelements, it is advantageous to find an alternative method to make their cultivation easier. Biochar can be a solution in this matter.

Biochar, defined as a material formed at high temperatures from 200 to 1000 °C as a result of pyrolysis, gasification, or hydrothermal/flash carbonization from carbon-rich substrates (agricultural residuals, biomass, or organic waste [14]), is perceived as soil improvement, showing a beneficial effect on its conditions and agricultural sustainability [15]. Its beneficial effects are demonstrated, among others, by improving the physical properties of the soil, such as its water-holding capacity and hydraulic conductivity. Additionally, there is an enhancement in porosity, bulk density, and aggregate stability [16]. Biochar also increases the retention of mineral nitrogen [17]. This material has a positive impact on plant cultivation by stimulating the activity of microorganisms present in the soil [18]. The reported increase in crop productivity also results from limiting the bioavailability of heavy metals [19,20], reducing the susceptibility of plants to water stress, resulting from salinity and high temperatures [21,22], and improving the uptake of nutrients by plants through their slow release [15]. Biochar application, as a material produced at high temperatures, eliminates the risk of accidental soil contamination with harmful microorganisms [10]. Importantly, biomass after pyrolysis becomes hydrophobic [23] and much easier to ground, resulting in easier storage and handling [24]. Moreover, this process also eliminates low-energetic compounds, leading to an increase in energy density per mass unit [25]. For this reason, biochar may be used as an alternative solid fuel in place of coal or other fossil fuels. Lignocellulosic biomass, such as agricultural residues, forestry residues, and herbaceous biomass, is primarily used for biochar production due to the high availability of these materials and the possibility of large-scale application [26]. For example, it was successfully implemented by Wang et al. [27], who investigated the production of biochar from corn stalk, corn cob, and spruce wood during slow pyrolysis at 600 °C. An even wider spectrum of substrates was used by He et al. [28], who generated biochar by slow pyrolysis at 300 °C and 600 °C from crop residues, i.e., wheat straw, rice straw, corn straw, rapeseed stalks, and cotton stalks. However, there is still not enough research on using flower waste to produce biochar, including the rose pruning biomass.

Currently, the search for new solutions in agriculture is closely related to the need to ensure its sustainable development. A major factor in this area is the concept of a circular economy, which aims to close the loops of raw materials and re-use waste to create regenerative systems. Importantly, achieving the global goal of agricultural system sustainability requires an understanding of the nature and potential applications of floral waste biomass [29]. The implementation of biochar produced from rose waste generated in horticultural farms is part of the objectives of the circular cycle by returning the carbon product to the soil as an organic fertilizer.

Therefore, the aim of this study was the characterization and evaluation of biochar produced from rose pruning waste for its application as a soil amendment. For this purpose, 21 types of biochar were produced, differing in their pyrolysis process conditions in terms of temperature (200–500 °C) and duration (20–60 min). The pollutant’s elution, heavy metal content, and phytotoxic effect of biochars were analyzed. Additionally, the fuel characteristics were evaluated.

## 2. Materials and Methods

### 2.1. Rose Pruning Waste

**Figure 1:**
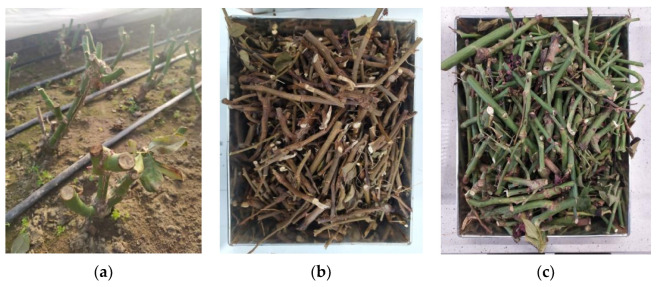
Spring pruning of roses: (**a**) plant seedlings after the treatment; (**b**) dry stem elements; and (**c**) green and semi-green waste of rose stems and roots.

For the production of biochar, waste from spring pruning (March–May) of large-flowered roses cultivated in the ground in foil tunnels at a horticultural farm in central Poland, Europe, was used. Dry, green, and semi-green stem fragments and plant roots were directed to the pyrolysis process (Figure 1).

### 2.2. Pyrolysis of Rose Pruning Waste

The biochar production process was carried out following the methodology specified in previous work [30]. The stems and roots of the rose were cut into pieces several centimeters in length using a garden pruner. Next, they were ground to a size of 1 mm using a laboratory knife mill (LMN-100, Testchem, Pszów, Poland). Then, the ground materials were placed in a muffle furnace (Snol 8.1/1100, Utena, Lithuania), where CO_2_ was supplied at 2.5 dm^3^ × min^−1^ to ensure inert conditions. The furnace was heated at a rate of 50 °C per min to reach the assumed temperature. The pyrolysis process was carried out at setpoint temperatures ranging from 200 to 500 °C with intervals of 50 °C. The residence time (time for keeping the set point temperature) for each temperature was 20, 40, and 60 min. Once the residence time was completed (counted from reaching a specific setpoint temperature), the furnace was turned off and allowed to cool down naturally. The CO_2_ was supplied until the temperature inside the furnace dropped below 200 °C. The temperature inside the reactor was controlled by a PID-type controller built into the furnace. To evaluate biochar production performance, the mass yield (MY), energy densification ratio (EDr), energy yield (EY), and energy gain (EG) were determined according to Equations (1)–(4):

(1) $$MY=\frac{m_{BC}}{m_{Raw}}\times 100$$

(2) $$EDr=\frac{{HHV}_{BC}}{{HHV}_{Raw}}\times 100$$

(3) EY=MY×EDr

(4) $$EG=\frac{({HHV}_{BC}-{HHV}_{Raw})/{HHV}_{Raw}}{(m_{Raw}-m_{BC})/m_{Raw}}\times 100$$

where MY—mass yield, %; $m_{BC}$—mass of produced biochar, g; $m_{Raw}$—mass of dry raw material, g; EDr—energy densification ratio, %; ${HHV}_{BC}$—a high heating value of produced biochar, $J\times g^{-1}$; ${HHV}_{Raw}$—a high heating value of dry raw material, $J\times g^{-1}$; EY—energy yield, %; and EG—energy gain, %.

### 2.3. Fuel Property Determination

The fuel properties of dry rose biomass and biochars were identified using proximate analysis and calorific value determination.

The proximate analysis included the determination of moisture content (MC), volatile matter (VM), fixed carbon (FC), and ash content (AC), which were all determined according to the PN-EN 14346:2011 standard [31] and thermogravimetric method [32,33]. Additionally, volatile solid (VS) content was determined according to the PN-EN 15169:2011 standard [34]. The calorific value analysis contained the determination of high heating values (HHVs) using a calorimeter (C200, IKA, Staufen, Germany), in accordance with the PN-EN ISO 18125:2017-07 standard [35].

### 2.4. Pollutant Elution and Heavy Metal Content

For all the produced biochar types and rose pruning waste (raw material), water extracts were made in triplicate [36]. The samples were prepared in a 1:10 ratio of solid to water (m/m): In 1000 mL sealed glass bottles, 80 g of biochar was placed, and 840 mL of purity grade 3 water was added. After 1 h, the flasks were closed and shaken for 4 h using a digital orbital shaker (ELMI DOS-20L, Calabasas, CA, USA). After unscrewing and storing the bottles for 16 h under static conditions, they were closed and shaken again for 4 h. For an additional 2 h, they were left for the sedimentation of the solids. The water extracts were then filtered using 0.45 µm filter paper and subjected to the physicochemical analyses listed in Table 1.

### 2.5. Phytotoxicity Tests

This experiment was carried out in triplicate using seed germination, and the early growth microbiotest with higher plants—Phytotoxkit [47]—with water extracts of biochars and raw material of rose pruning waste was conducted according to the procedure described in Section 2.3. Additionally, distilled water was used instead of the biochar water extracts to serve as a control sample.

First, a test of the water-holding capacity of the reference soil was performed. After sifting through a 2 mm mesh sieve, 90 cm^3^ of material was placed in a 100 cm^3^ cylinder. Then, 50 cm^3^ of distilled water was added and mixed until the soil was completely saturated. After reaching the equilibrium state, the supernatant was poured into a 50 mL measuring cylinder. This activity was repeated until a layer of water did not precipitate above the surface of the material.

The volume of water required to completely saturate the reference soil, corresponding to the difference in the volume of water added to the soil and the volume of the supernatant recovered in the measuring cylinder, was calculated based on the following equation:

(5) $$V_{sat}=50-S$$

where *V_sat_*—water-holding capacity, cm^3^ and *S*—volume of the supernatant, cm^3^.

**Figure 2:**
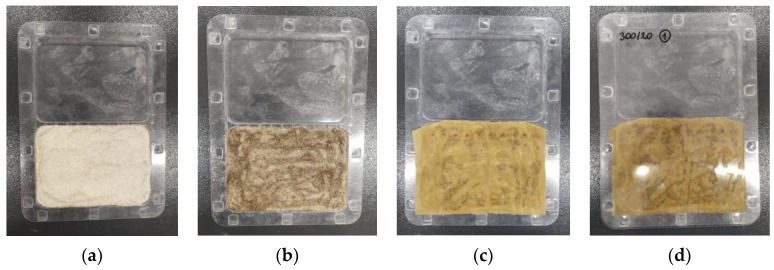
The procedure of using the Phytotoxit test: (**a**) reference soil on a test plate; (**b**) soil saturation with distilled water; (**c**) *Lepidium sativum* seeds on a paper filter; and (**d**) a closed test plate prepared to incubation.

Next, the 90 cm^3^ of reference soil was placed on a transparent PVC test plate composed of a bottom part separated by a middle ridge into two compartments and a flat cover (Figure 2). The material was aligned to create a flat layer of even thickness. Then, 30 cm^3^ of water extracts were introduced into the soil with a syringe. A paper filter was placed on the surface of the soil, and at its top edge, 10 seeds of *Lepidium sativum* were placed in one row at the same intervals. The closed plate was vertically incubated for 3 days in the dark at 25 °C in a climate chamber (ST 3 BASIC, POL-EKO-APARATURA, Wodzisław Śląski, Poland). Photographic documentation of the seed germination capacity was recorded with a camera after 3 days, and the root length was measured using ImageJ software (https://imagej.net/ij/, accessed on 12 April 2024), Wayne Rasband.

The percentage of seed germination was calculated according to Equation (6):

(6) $$A=100\times \frac{\left(a-b\right)}{a}$$

where *A*—seed germination, %; *a*—total amount of seeds; and *b*—number of non-sprouted grains.

The inhibition of root elongation was calculated based on Equation (7):

(7) $$I=100\times \frac{\left(C-T\right)}{C}$$

where *I*—inhibition of root elongation, %; *C*—the root length value measured for samples with the reference soil, mm; and *T*—the value of the root length measured for samples with the tested material, mm. Data on the percentage of seed germination and inhibition of root elongation were analyzed using Statistica StatSoft Inc. (Tulsa, OK, USA), TIBCO Software Inc. (Palo Alto, CA, USA), for estimating the measurements’ mean and standard deviation.

## 3. Results and Discussion

### 3.1. Pyrolysis of Rose Residues and Biochar Production

Table 2 shows photographic documentation of all biochar produced by pyrolysis for different temperatures (200–500 °C) and process durations (20–60 min).

Table 3 shows the effect of pyrolysis temperature and process duration on the mass and energy yield, energy densification ratio, and energy gain of biochar. With the increase in pyrolysis temperature from 200 °C to 500 °C, the mass yield of biochar gradually decreased from 97.7 ± 0.2 to 32.0 ± 0.5%. This tendency can be connected with the decomposition of the biomass constituents, such as hemicellulose, cellulose, and lignin, into noncondensable gaseous products (e.g., CO, CO_2_, H_2_O, and CH_4_) and liquid bio-oil products (e.g., acids, aldehydes, ketones, and phenols) [48]. Cellulose degradation occurs within the temperature range of 200 °C to 260 °C, hemicellulose degradation occurs between the temperatures of 200 °C and 260 °C, and lignin decomposes at temperatures of 280 °C to 500 °C [49]. The energy yield of biochar decreased with increasing temperature and process duration from 98.9 ± 0.8 to 48.0 ± 0.7% due to the loss of volatile substances [50]. At all temperatures, the energy yield of biochar was greater than the mass yield. The energy densification ratio increased under higher temperatures, from 1.01 ± 0.00 at 200 °C to 1.50 ± 0.00 at 500 °C. This indicates that an increased energy densification ratio at a lower mass leads to a higher-quality product. The process duration influenced the production parameters of biochar. For instance, higher mass and energy yields were observed when using a temperature of 350 °C and a process duration of 20 min, compared to a temperature of 300 °C and a process duration of 60 min. The highest energy gain, reaching 92.5 ± 14.8%, was observed for a temperature and process duration of 250 °C for 40 min, while the lowest, reaching 30.7 ± 5.5% also at 250 °C, was characteristic for a process duration of 20 min.

The results presented here for biochars derived from rose pruning waste are consistent with values reported in the literature. Torres-Sciancalepore et al. [51] used slow pyrolysis to obtain biochar from *Rosa rubiginosa* seed waste. The biochar mass yields obtained by these researchers at 270 °C, 330 °C, and 400 °C were equal to 74.32%, 44.79%, and 34.20%, respectively [51]. Additionally, Cifuentes et al. [52] reported that the mass yield of activated carbon from rose stems pyrolyzed at 500 °C, 600 °C, and 700 °C were 37.5 ± 0.5%, 34.4 ± 0.6%, and 30.8 ± 0.5%, respectively [52]. Mokrzycki et al. [53] reported that the energy densification ratio and energy yield of black alder cone-like flowers (*Alnus glutinosa* L. Gaertn.) at 250 °C, 300 °C, 400 °C, and 500 °C were 1.34, 1.42, 1.58, 1.70, and 96%, 81%, 68%, 63%, respectively [53].

### 3.2. Biochar’s Fuel Properties

The results of the proximate analysis and calorific values for all samples are presented in Table 4. In general, the volatile matter (VM) and volatile solids (VSs) decreased with increasing temperature. In contrast, as the temperature grew, the fixed carbon (FC) and ash content (AC) gradually increased. The highest VSs were observed for raw rose pruning waste and biochar produced at 200 °C for 40 min (both of which reached 97.2 ± 0.4%). Similarly, the highest VM was again characteristic for raw rose pruning biomass, reaching 75.5 ± 0.5%. A high content of volatile matter at relatively low temperatures is due to the presence of lignin in feedstock. Lignin can resist pyrolytic decomposition at 400 °C but not at temperatures as high as 950 °C [54]. The lowest FC and AC were recorded for raw rose pruning waste with values of 21.7 ± 0.7% and 2.8 ± 0.4%, respectively. The same ash content was also recorded for biochar at 200 °C for 40 min. On the other hand, at 500 °C, the biochar had the lowest VSs (85.9 ± 2.1%) and the highest AC (14.1 ± 2.1%). The minimum VM and the maximum FC were observed at 450 °C, reaching 25.0 ± 2.0% and 63.5 ± 3.1%, respectively. The increase in fixed carbon and ash content with temperature can be caused by the removal of volatile matter, leaving the more stable carbon and ash-forming inorganic matter in biomass [55]. During pyrolysis, heavy metals, like chromium (Cr), nickel (Ni), copper (Cu), zinc (Zn), arsenic (As), cadmium (Cm), and lead (Pb), accumulate in the ash fractions [56]. This is important, since the ash content present in soil has a significant impact on the growth of plants. Dai Y. et al. [57] found no significant differences in plant growth when the ash content of biochar was <10%. Plant growth was found to be positively impacted by ash contents greater than 10% and as high as 25% [57]. The high heating value of biochar (HHV) represents the quantity of energy that can be obtained from the combustion of biomass [58]. The highest measured HHV was 27,443 ± 168 J × g^−1^ under a 500 °C pyrolysis temperature, while the lowest was 18,312 ± 235 J × g^−1^ for raw rose pruning waste.

In previous studies for biochar produced from other crop residues, it was found that the distribution of volatile matter, ash content, fixed carbon, and high heating values was similar to the results presented in this manuscript. For example, Mokrzycki et al. [53] produced biochar from black alder cone-like flowers (*Alnus glutinosa* L. Gaertn.). Their initial input material was characterized by 84.2 ± 0.2% of VM, 2.6 ± 0.1% of AC, 29.6 ± 0.2% of FC, and 15,900 ± 200 J × g^−1^ of HHV. After the pyrolysis at 500 °C, their biochar’s properties changed roughly to 16.8 ± 0.3% of VM, 5.8 ± 0.5% of AC, 76.6 ± 0.3% of FC, and 27,200 ± 200 J × g^−1^ of HHV, respectively [53]. Keiluweit M. et al. [59] reported that the VM, AC, and FC of tall fescue grass (*Festuca arundinacea*) at 200 °C were 70.7% VM, 5.7% AC, and 23.6% FC, respectively. However, after subjecting their feedstock to a 500 °C pyrolysis temperature, their values changed to 20.3% VM, 15.4% AC, and 64.3% FC, respectively [59].

### 3.3. Pollutant Elution

The concentration of pollutants in the water extracts of biochars varied depending on the applied temperature and duration of the pyrolysis process (Table 5). In general, biochars were mostly characterized by a lower total carbon content than the raw material (with two exceptions for the biochars produced at 200 °C and 500 °C for 60 min, where their values exceeded 4840 ppm and 4680 ppm, respectively). The total carbon content and organic carbon content varied greatly between the variants, reaching extreme values for two samples: 400 °C for 40 min and 200 °C for 60 min (50 ± 3 ppm vs. 4840 ± 61 ppm for total carbon content and 94 ± 2 ppm vs. 4920 ± 61 ppm for organic carbon content, respectively). The rose pruning waste and biochar variants of 500 °C for 60 min achieved the same amount of total carbon content, organic carbon content, and inorganic carbon content (4680 ± 18 ppm, 4760 ± 25 ppm, and 80 ± 7 ppm, respectively). After the pyrolysis process, the inorganic carbon content of biochars had a narrow range of values from 48 ± 2 ppm at 350 °C to 183 ppm at 450 °C. Kjeldahl nitrogen and organic nitrogen content were similar for samples produced in all pyrolysis temperatures and duration. While the values of organic nitrogen were mostly in line with the findings of other authors, the observed values of total carbon content were lower. Anyikude [60] produced biochar from greenhouse waste, green waste, and pig manure at 400 °C. The organic carbon content of their biochar water extracts was 4610 ppm, 1331 ppm, and 3584 ppm, and that of their organic nitrogen was 49 mg N_org_·dm^−3^, 53 mg N_org_·dm^−3^, and 267 mg N_org_·dm^−3^, respectively [60]. The concentration of ammonical nitrogen in biochar decreased with increasing temperature, from 54.49 N_NH4_·dm^−3^ at 200 °C for 20 min to 0.02 N_NH4_ × dm^−3^ at 400 °C for 60 min. For temperatures above 350 °C, the values of ammonical nitrogen were <1 N_NH4_ × dm^−3^. The amount of nitric nitrogen varied depending on the temperature and process duration, ranging from 0.15 N_NO3_ × dm^−3^ at 500 °C for 40 and 60 min to 101.1 N_NO3_ × dm^−3^ at 250 °C for 60 min. The nitric nitrogen content of <1.0 N_NO3_ × dm^−3^ was observed for all temperatures above 450 °C. Comparing the process conditions of 200 °C for 20 min and 500 °C for 60 min, the total nitrogen content of the biochar decreased by 79.3%. Total nitrogen loss in biochar is attributed to evaporative denitrification [61]. When it comes to its availability in soil, according to Ye Z. et al. [62], the decomposition of nitrogen mass is influenced by the nitrogen retention rate of biochar, which decreases with increasing pyrolysis temperature [62]. Additionally, increasing the release of volatile compounds and acidic functional groups at high temperatures may also impact the release of nitrogen from biochar. In addition, under high pyrolysis temperatures, heterocyclic ammonium nitrogen, like pyrrole and pyridine, can cause low nitrogen availability, making it less absorbable by plants [63]. Since they absorb nitrogen from the environment to construct their structures and perform cellular functions, these effects are crucial.

At lower pyrolysis temperatures, the total phosphorus content was generally higher and gradually decreased with an increase in temperature (from 4.6 mg P × dm^−3^ at 400 °C for 60 min to 135.8 mg P × dm^−3^ at 250 °C for 40 min). As the total phosphorus content of soil typically ranges from 0.2 to 1.2 g × kg^−1^, various feedstocks can increase soil stock after pyrolysis [64]. By releasing phosphorus directly into the soil, the application of biochar increases the availability of some of its native soil forms and adds to the overall content of this element in this medium [64]. Based on these test results, phosphorus-rich biochar can be generated under the specific pyrolysis process conditions with a low temperature (max. 350 °C).

The dissolved oxygen content increased at higher temperatures from 0 mg O_2_ × dm^−3^ for the raw rose pruning material to 6.2 mg O_2_ × dm^−3^ at 500 °C for 20 min. The biological oxygen demand (BOD_5_) was notably higher than that reported by other researchers [65]. The obtained values ranged from 45 mg O_2_ × dm^−3^ at 500 °C for 60 min to 3048 mg O_2_ × dm^−3^ for raw rose pruning waste (Table 5). Different concentrations of metal ions, such as Al, Co, Ni, Cu, Zn, Pb, and Hg, in biochar extracts may be the reason for the observed differences since their addition can both lower or increase the BOD_5_, depending on their concentration [66]. The value of chemical oxygen demand (COD) represents the amount of oxygen required to oxidize organic compounds [67]. According to the results, the COD value was associated with the content of organic compounds found in water extracts, such as organic carbon and organic nitrogen. A larger concentration of oxygen is needed to oxidize the organic compounds found in greater concentrations in the aqueous extracts. The value of general suspensions under each of the pyrolysis process temperatures (except at 450 °C and 500 °C) was highest with a process duration of 20 min, reaching from 1610 mg × dm^−3^ to 62 mg × dm^−3^. Similarly, the value of general dissolved substances at each of the tested temperatures was highest for a process duration of 20 min with the exception of the 250 °C and 500 °C variants. It ranged from 9235 mg × dm^−3^ to 517 mg × dm^−3^. In general, with the increase in temperature and duration, the value of pH gradually increased, reaching a maximum of 10.8 at 450 °C and 500 °C for 60 min variants. This may be related to the elevated relative concentration of non-pyrolyzed inorganic elements in the feedstocks and the generation of basic surface oxides under high pyrolysis temperatures [68].

The acidic pH of biochar produced at lower temperatures is due to the presence of acidic functional groups, most of which are removed when the temperatures are high [69]. The pH of biochar is one of the key properties that can significantly affect plant growth [57]. According to Dai Y et al. [57], the use of a biochar amendment with a pH < 7 showed a significant decrease in plant growth. In contrast, compared to the other groups tested, the biochar samples with pH 7–8 showed the greatest increase in plant growth. For all the tested samples (Table 5) the biochars produced at 350 °C (for 40 and 60 min), 400 °C for 60 min, and 450 °C for 20 min have the most optimal material for plant growth in terms of their pH value. On the other hand, the least favorable biochars, in this regard, were those produced at 200 °C, 250 °C, 300 °C (for each duration), and 350 °C for 20 min. The observed values of electrical conductivity (EC) were consistent with the results of other authors. Bachmann et al. [70] reported that the electrical conductivity for a blend of paper sludge and wheat husks at 500 °C was 1054 μS × cm^−1^ [61]. Kloss et al. [71] reported that at 400 °C, the value of conductivity for biochars of wheat straw was 1080 μS × cm^−1^, 1040 μS × cm^−1^ for poplar (*Populus tremula*) wood, and 420 μS × cm^−1^ for spruce (*Picea abies*), respectively [71]. At 460 °C, the values were 4920 μS × cm^−1^ for wheat straw, 700 μS × cm^−1^ for poplar wood, and 1810 μS × cm^−1^ for spruce, respectively [71]. Generally, the value of biochar EC relies heavily on the presence of soluble OH groups and soluble monovalent cations, primarily potassium [70]. 

### 3.4. Heavy Metal Content

The heavy metal contents of the biochar extracts produced at different temperatures and process duration variants are illustrated in Table 6. The amount of metals in biomass, including alkali metals, alkaline earth metals, and heavy metals, depends on the biomass type, growth conditions, and the geographical location of the region [72]. Heavy metals and metalloids contained in the original feedstock, such as Cd, Pb, and Hg, may undergo volatilization during the pyrolysis process or be concentrated in the biochar [70]. The concentrations of all tested metals, except for cadmium and lead, were higher in the raw material water extracts compared to the extract of biochar produced at 500 °C for 60 min (Table 6). In the raw material, the highest values were found for potassium (504.4 mg K × dm^−3^) and calcium (71.7 mg Ca × dm^−3^), while no contents of cadmium and lead were noted. During the pyrolysis process conducted at 500 °C for 60 min, biochar extracts contained the highest value of potassium (315.2 mg K × dm^−3^) and sodium (12.7 mg Na × dm^−3^); again, no cadmium or lead were found in these samples. On the other hand, the metal content of the biochar extracts was higher at process temperatures of 200 °C, 250 °C, and for some metals at 300 °C compared to the raw material. However, an increase in temperature above 300 °C or 350 °C lowered the metal content of the biochar extracts. The same trend of decreasing the heavy metal contents of biochars with increasing pyrolysis temperature was observed by Larina et al. [73], who studied the solubility of heavy metals in sewage sludge before and after the pyrolysis process at 250 °C and 800 °C. The metal contents of the biochar extracts also changed at different process durations. This tendency matched the findings of Chandra et al. [74], who obtained different values for the metal contents of their biochar extracts depending on the duration (60, 90, and 120 min) of the pyrolysis process (400–700 °C). For example, for the extracts of the samples produced at 300 °C for 20 min, the potassium content was 677.9 mg K × dm^−3^, but extending the process duration to 40 min reduced its content by more than half (331.3 mg K × dm^−3^) (Table 6). Agricultural soils are frequently contaminated by heavy metals and metalloids, such as Cd, Pb, Cr, As, Hg, Ni, Cu, and Zn, which can be harmful to plants at high concentrations. The most highly toxic and harmful metals to plant health across nearly all defilement levels are Cd, Pb, As, Hg, and Cr. Inversely, Cu, Zn, Fe, Mn, Mo, Ni, Mg, Ca, and B, at relatively low concentrations, can enhance particular cellular capacities in plants, including ion homeostasis, pigment biosynthesis, photosynthesis, respiration, enzyme activities, gene regulation, sugar metabolism, and nitrogen fixation. However, if these elements accumulate in concentrations above the optimum or fall below a certain threshold level, they will cause adverse effects on plant growth, development, and reproduction [75].

According to the national regulation for fertilizers and plant growth enhancers in Poland [76], all of the tested biochar and raw materials did not exceed the permissible values for heavy metals, including Cr, Cd, Ni, and Pb. Moreover, the contents of heavy metals (Table 6) were much lower than those regulations limits (for Cr: 0.5 mg × kg d.m.^−1^ vs. 100 mg × kg d.m.^−1^; for Cd: 0.01 mg × kg d.m.^−1^ vs. 5.0 mg × kg d.m.^−1^; for Ni: 0.6 mg × kg d.m.^−1^ vs. 60 mg × kg d.m.^−1^; and for Pb: 0.0 vs. 140 mg × kg d.m.^−1^) [76]. Therefore, in terms of heavy metal content, all of the tested biochar samples and raw materials are safe to be used as organic fertilizers.

### 3.5. Phytotoxicity Tests

Table 7 shows the results of the phytotoxicity test for biochars derived from rose pruning waste. In general, the extracts of biochars produced at higher temperatures with a longer process duration caused an increase in the average root length. The highest value was observed at 450 °C for 40 min, while the lowest average root length occurred at 200 °C for 20 min (72.16 ± 14.67 and 17.08 ± 3.56 mm, respectively). In general, the biochars produced at a temperature ≥400 °C did not inhibit root elongation (with the inhibition index ranging from −3.45 ± 20.12 to −31.68 ± 14.67% for the biochar variants at 500 °C for 60 min and 450 °C for 40 min, respectively); the only exception was the material prepared at 450 °C for 60 min (4.08 ± 23.34%). Among the biochars from the pyrolysis process at lower temperatures, the biochar variant at 250 °C for 60 min was the only one that also showed a positive effect on root elongation (−1.59 ± 15.84%). In most cases, the average seed germination was high (>80%). Lower values were noted for the biochars generated at 200 °C (all process duration variants; average seed germination from 13.33 ± 3.56 to 73.33 ± 14.46% for 20–60 min, respectively) and 250 °C (biochar produced for 20 min). The obtained results were consistent with the study conducted by Rombolà et al. [77], who tested the phytotoxicity of biochar made from poultry litter (PL) and corn stalks (CSs) at 400 °C and 500 °C using cress (*Lepidium sativum* L.) germination tests [77]. The results of relative seed germination were as follows: 83 ± 4% PL and 98 ± 2% CS for 400 °C and 77 ± 2% PL and 97 ± 5% CS for 500 °C [77]. Similar results of the average root length and value of seed germination (Table 7) were also obtained by Gezahegn et. al. [78], who studied the phytotoxicity of biochar from leachate at 300–700 °C produced by slow pyrolysis using radishes (*Raphanus raphanistrum* subsp. sativus) [68]. The results of the average root length and average seed germination were ~40–50 mm and ~95%−100%, respectively [78]. The increase in seed germination with the amendment of biochars produced at higher temperatures can be attributed to the alkalinity of the pyrolysis product. It can be explained by the increase in alkaline elements’ concentration, such as calcium and magnesium, and the simultaneous fall in the acidic components’ level. It has been observed that alkaline solutions can enhance seed germination by eliminating natural inhibitors of germination, such as abscisic acid [78].

## 4. Conclusions

This study showed that it is most recommended to produce rose pruning waste biochar at 300 °C for 40 and/or 60 min to obtain an alternative solid fuel with the best properties. These specific process conditions result in high energy gains (77.6 ± 1.5% and 74.8 ± 1.5%), energy densification ratios (1.35 ± 0.00 and 1.37 ± 0.00), high heating values (24,720 ± 267 J × g^−1^ and 25,113 ± 731 J × g^−1^), and optimal ash contents (5.9 ± 0.5% and 7.1 ± 0.3%), competitive to other biochars that have been tested. Although the material produced at 250 °C for 40 min showed the highest energy gain (92.5 ± 14.8%) among all variants, the high heating value was lower (21,463 ± 396 J × g^−1^). Therefore, these process conditions are not recommended.

Regarding using biochars as soil amendments, the produced samples demonstrated better fertilizing properties compared to the raw material. Rose pruning waste was characterized by an acidic pH value (5.5), higher content of heavy metals, such as zinc (1464.0 μg Zn × dm^−3^), copper (312.5 μg Cu × dm^−3^), or chromium (133.0 μg Cr × dm^−3^), and higher pollutant content, such as general suspensions (1865 mg × dm^−3^), compared to biochars. In contrast, all of the tested biochars did not exceed the permissible values for heavy metals, including Cr, Cd, Ni, and Pb. Generally, biochars that were produced at a temperature ≥400 °C did not inhibit root elongation, except for the material produced at 450 °C for 60 min (4.08 ± 23.34%). Biochars obtained at ≥300 °C showed a positive impact on seed germination (86.67 ± 18.48–100 ± 24.14%). For the best fertilizer effects, it is recommended to produce biochar at 400 °C for 40 min, 450 °C for 20 min, and 500 °C for 20 min. These material variants are characterized by their optimal pH values (8.6, 7.0, and 8.4, respectively), low values of heavy metals content (such as Zn, Cu, Cd, Pb, Cr, Mn, and Fe), and relatively high ash content (9.0 ± 2.7%, 9.3 ± 2.1%, and 12.2 ± 1.9%, respectively) compared to the other samples tested. Moreover, the values of the average root length occurred at a high level (64.16 ± 16.79 mm, 59.61 ± 20.48 mm, and 57.95 ± 24.47 mm, respectively), and no inhibition of root elongation occurred.

To sum up, the idea of closing the loop by using biochar produced from rose pruning waste as an alternative solid fuel and returning it to crops as a soil amendment is an important element of our circular economy. This study confirmed the produced biochars’ applicability in both scenarios. However, it is necessary to carefully select the pyrolysis process conditions to obtain valuable and safe material.

## Figures and Tables

**Table 1:** Analyses of pollutants in water extracts.

Pollution Indicator	Determination Method
Total organic carbon (TOC), total carbon (TC), and total inorganic carbon (IC)	Sievers InnovOx Labolatory TOC analyzer (GE, Analyttical Instruments, General electric, Co., Ltd., Boston, MA, USA)
Total nitrogen, mg N × dm^−3^	The sum of all forms of nitrogen
Kjeldahl nitrogen, mg N × dm^−3^	PN-EN 25663:2001 [37]
Organic nitrogen, mg N_org_ × dm^−3^	Indirectly (difference between Kjeldahl and ammonium nitrogen)
Ammoniacal nitrogen, mg N_NH4_ × dm^−3^	PN-C04576-4:1994 [38]
Nitric nitrogen, mg N_NO3_ × dm^−3^	PN-C-04576-08:1982 [39]
Total phosphorus, mg P × dm^−3^	PN-EN 1189-2000 [40]
Dissolved oxygen, mg O_2_ × dm^−3^	PN-EN ISO 5814:2013-04E [41]
BOD_5_, mg O_2_ × dm^−3^	PN-EN 1899-1:2002 [42]
COD (Cr), mg O_2_ × dm^−3^	PN ISO 15705:2005 [43]
General dissolved substances, mg × dm^−3^	By weight, after the evaporation of the filtered sample
General suspensions, mg × dm^−3^	PN-EN 872:2007 [44]
pH	PN-EN ISO 9963-1:2001 [45]
Conductivity, μS × cm^−1^	PN-EN 27888:1999P [46]
Sodium, mg Na × dm^−3^	Atomic emission spectrometry (AAS, according to standards provided by Spectro-Lab Ltd., Los Angeles, CA, USA)
Potassium, mg K × dm^−3^	
Calcium, mg Ca × dm^−3^	
Magnesium, mg Mg × dm^−3^	
Zinc, μg Zn × dm^−3^	
Copper, μg Cu × dm^−3^	
Nickel, μg Ni × dm^−3^	
Cadmium, μg Cd × dm^−3^	
Chromium, μg Cr × dm^−3^	
Manganese, mg Mn × dm^−3^	
Iron, mg Fe × dm^−3^	
Lead, μg Pb × dm^−3^	

**Table 2:** Photographic documentation of all biochar samples.

	20 min	40 min	60 min
200 °C	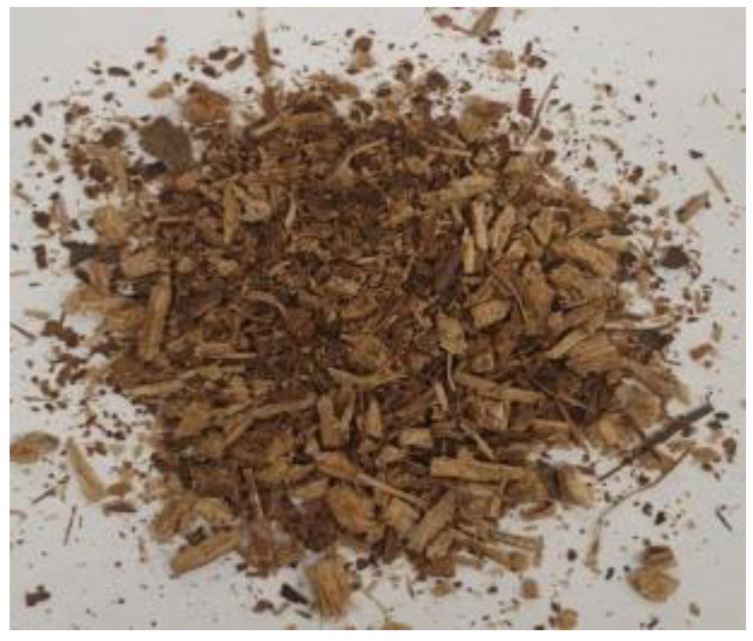	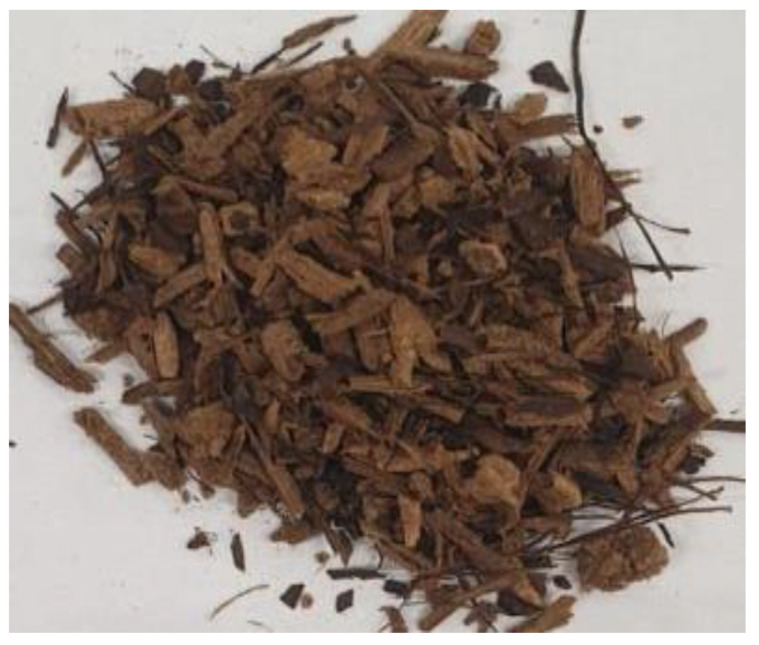	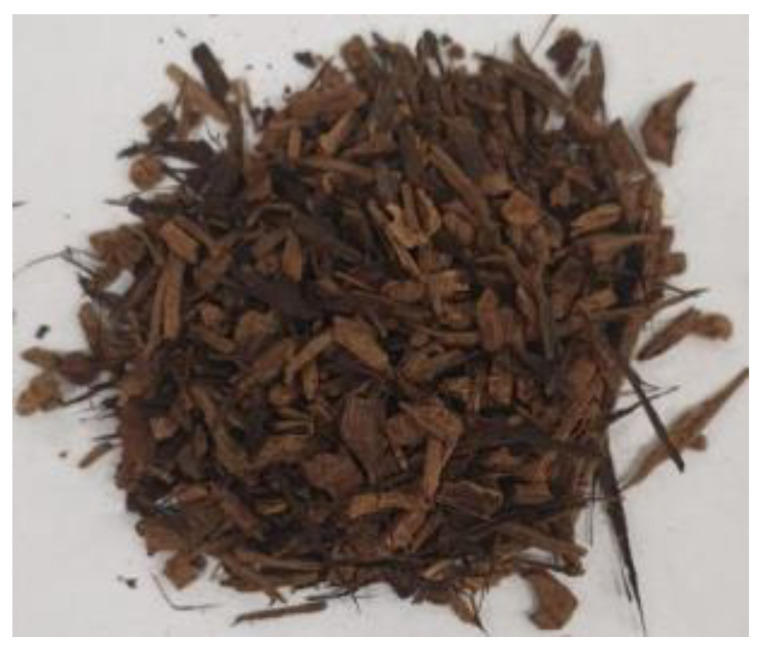
250 °C	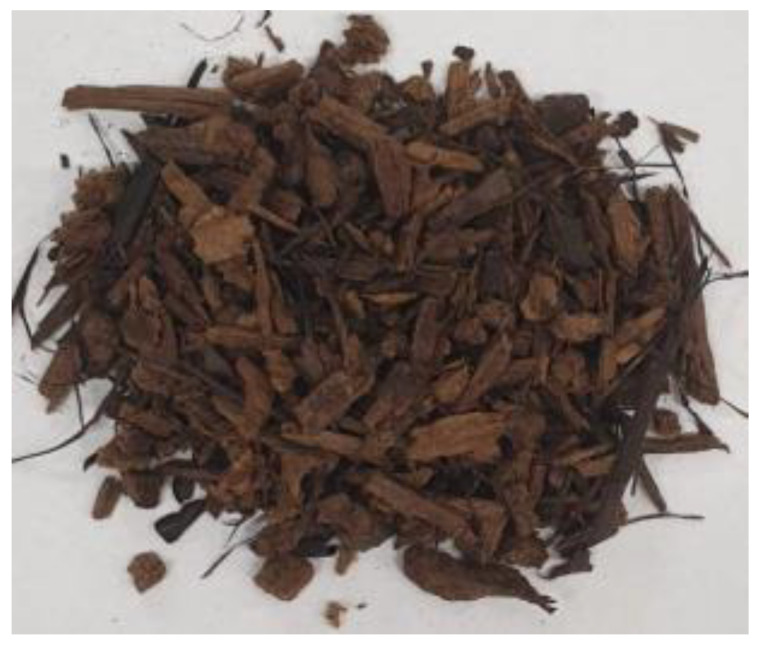	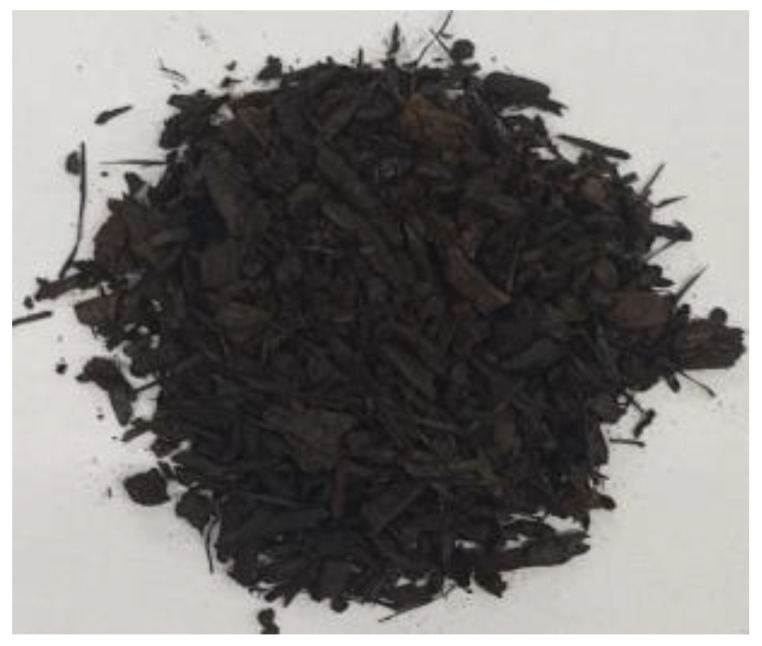	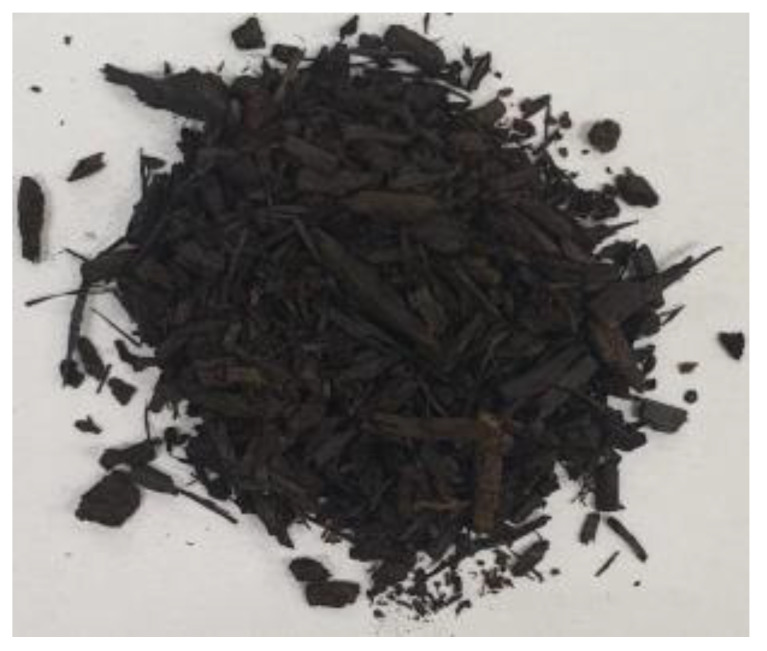
300 °C	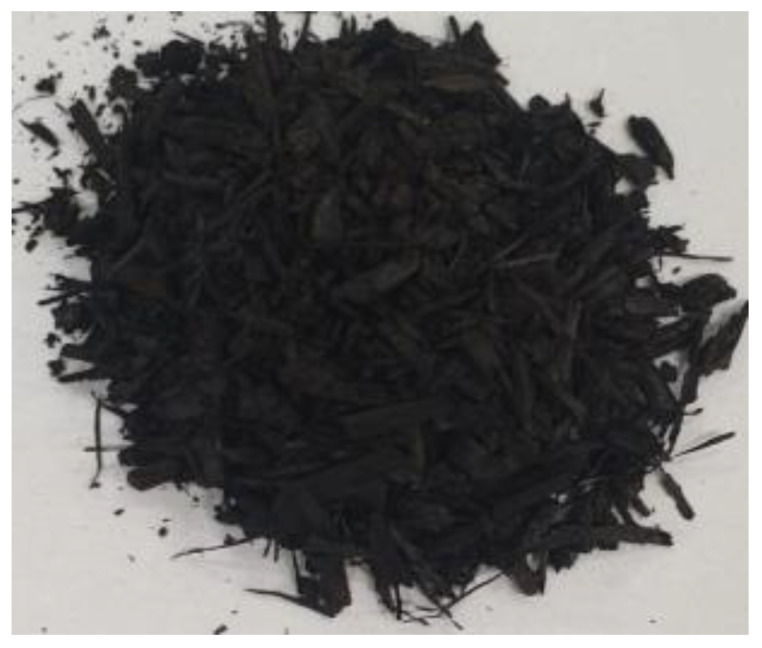	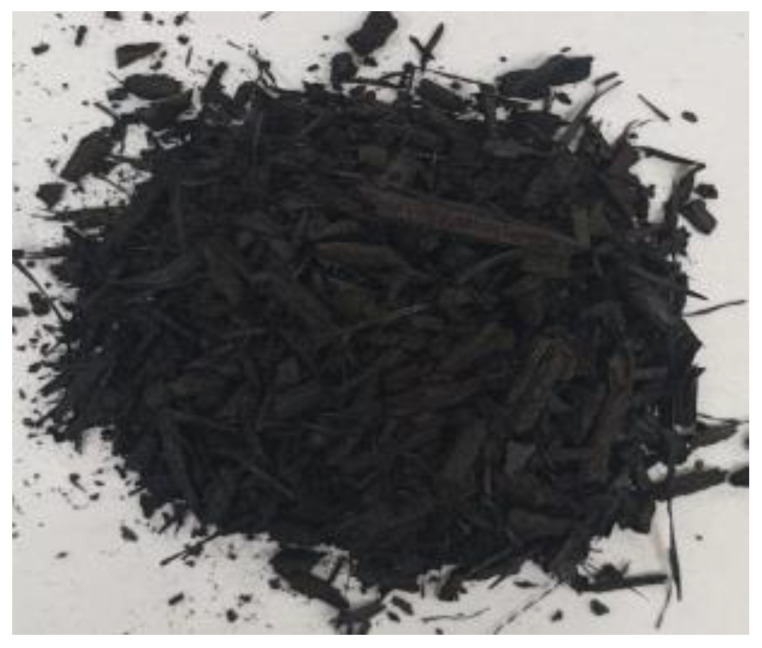	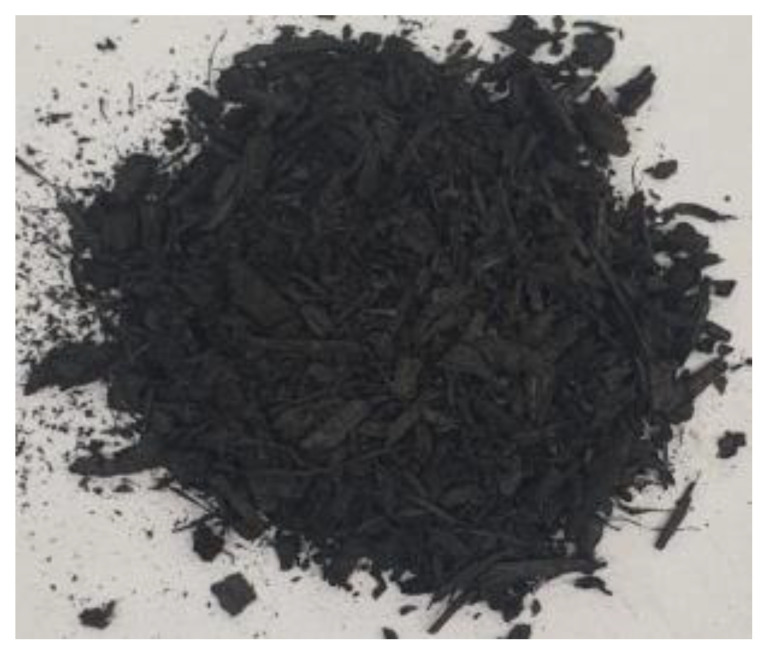
350 °C	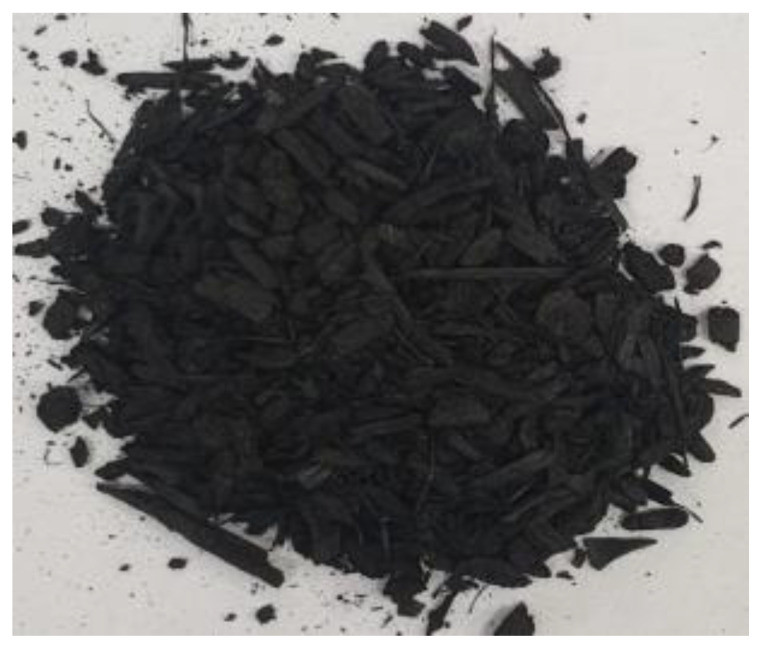	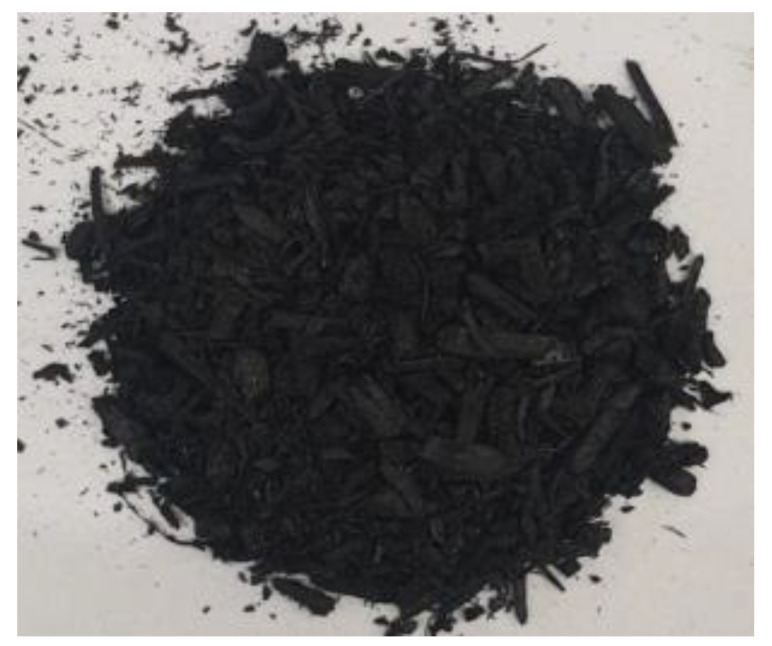	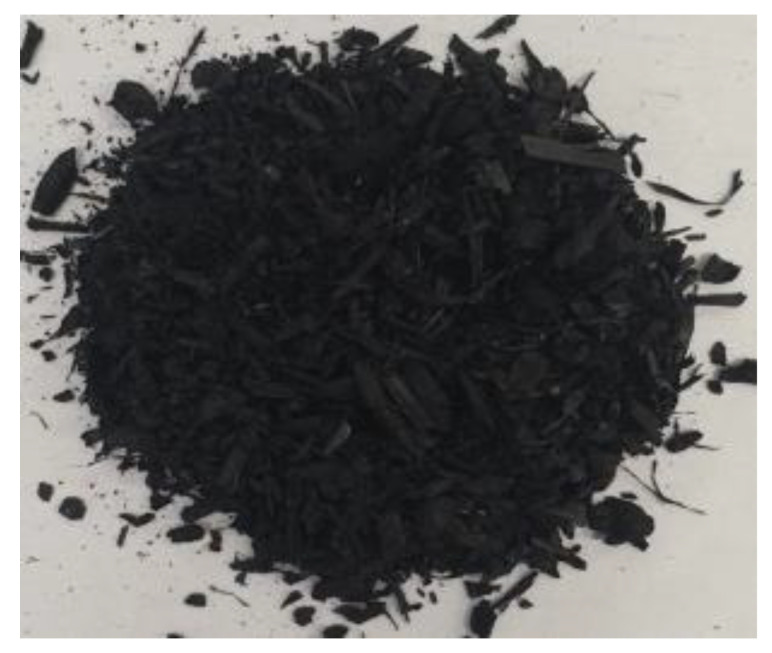
400 °C	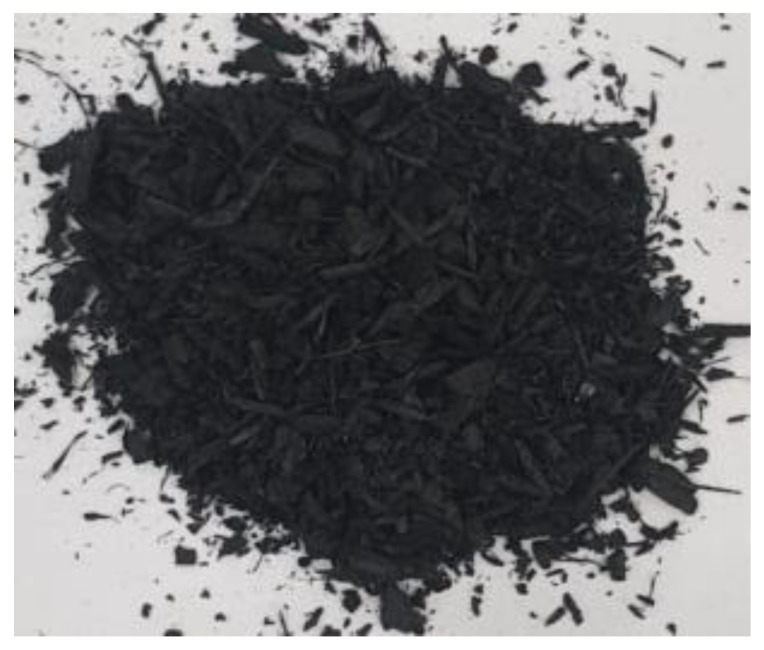	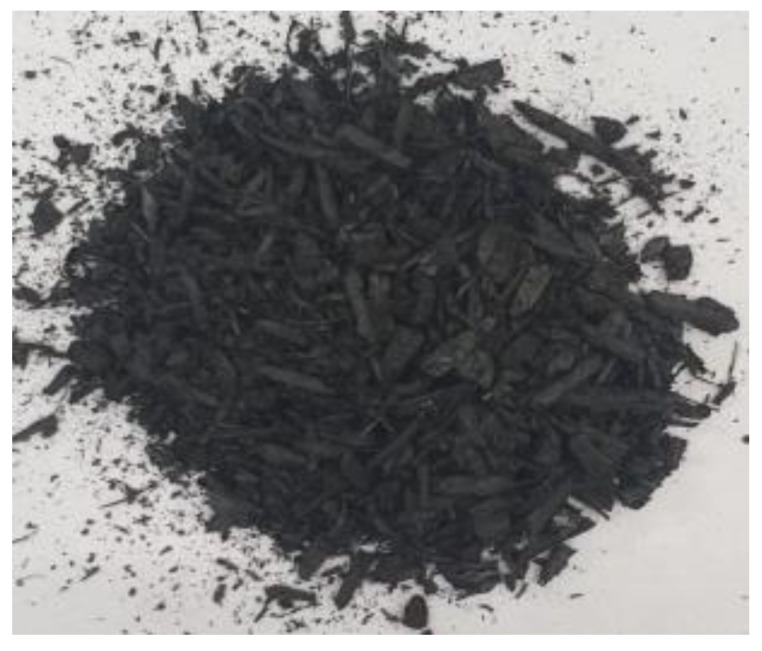	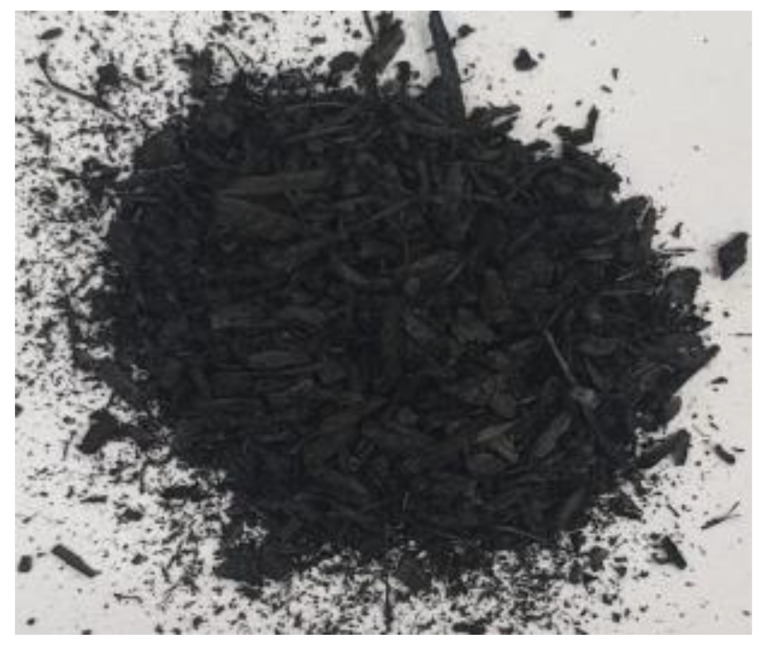
450 °C	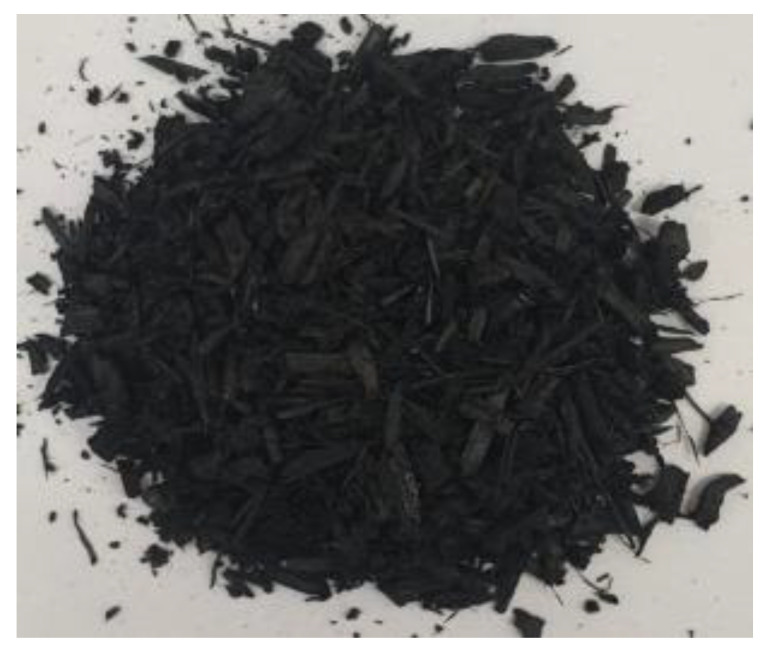	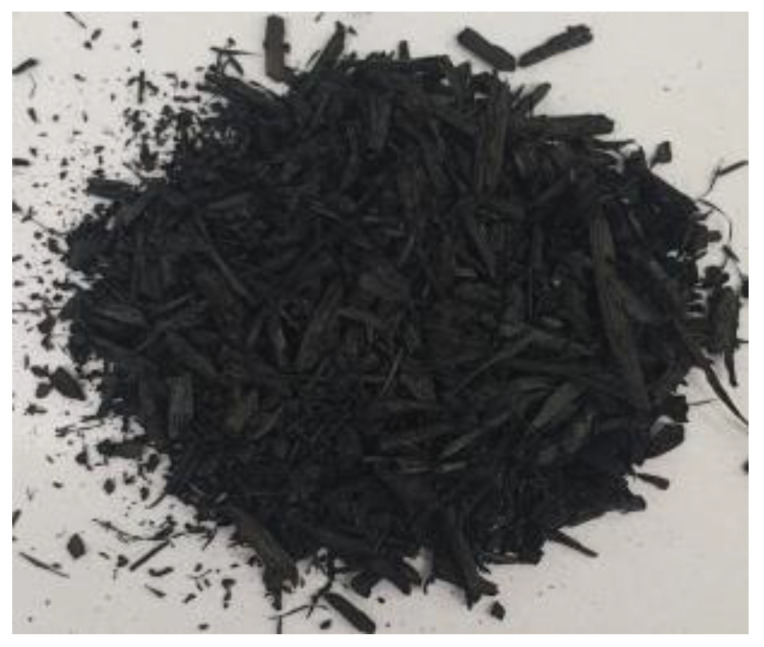	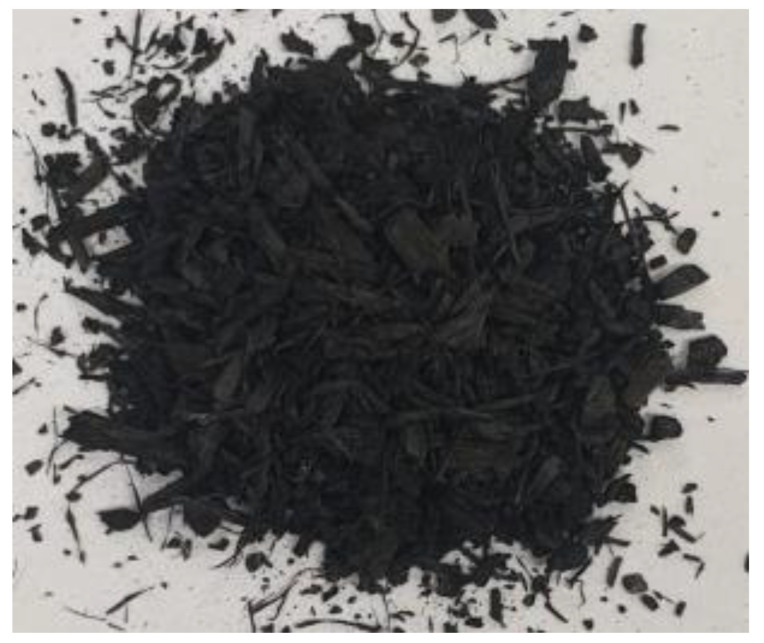
500 °C	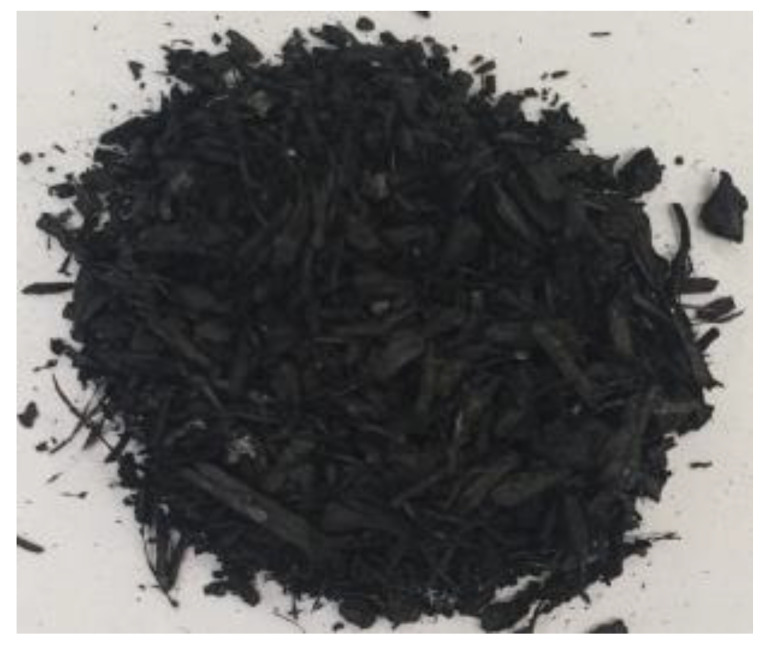	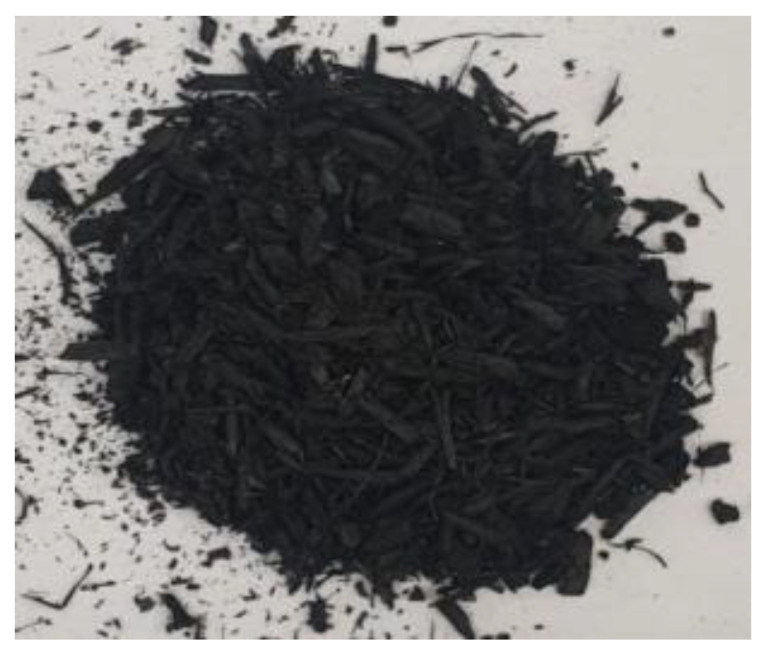	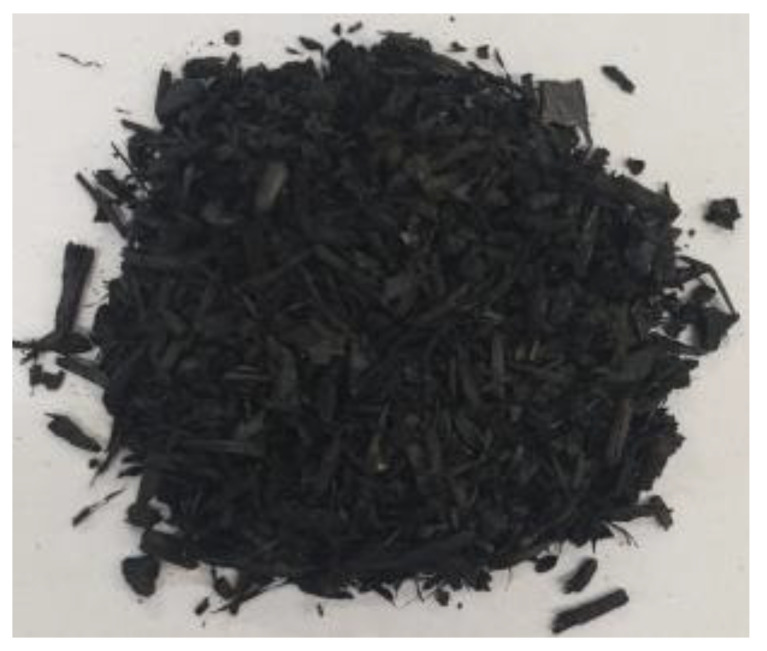

**Table 3:** Biochar production performance parameters. The raw data can be found in the Supplementary Materials (sheet: ‘BC production’).

T, °C	t, min	MY, %	EDr, -	EY, %	EG, %
200	20	97.7 ± 0.2	1.01 ± 0.00	98.6 ± 0.2	41.2 ± 3.1
	40	96.2 ± 0.8	1.03 ± 0.00	98.9 ± 0.8	74.9 ± 14.9
	60	92.1 ± 0.3	1.06 ± 0.00	97.5 ± 0.4	74.1 ± 3.1
250	20	92.9 ± 1.3	1.02 ± 0.00	94.9 ± 1.3	30.7 ± 5.5
	40	81.0 ± 3.0	1.17 ± 0.00	95.0 ± 3.5	92.5 ± 14.8
	60	72.9 ± 0.6	1.18 ± 0.00	86.1 ± 0.7	66.8 ± 1.5
300	20	77.3 ± 1.5	1.10 ± 0.00	84.8 ± 1.7	43.2 ± 2.9
	40	54.9 ± 0.9	1.35 ± 0.00	74.1 ± 1.2	77.6 ± 1.5
	60	50.4 ± 1.0	1.37 ± 0.00	69.1 ± 1.3	74.8 ± 1.5
350	20	56.2 ± 3.4	1.26 ± 0.00	70.8 ± 4.3	59.9 ± 4.7
	40	45.4 ± 3.0	1.36 ± 0.00	61.6 ± 4.1	65.7 ± 3.8
	60	43.5 ± 0.4	1.38 ± 0.00	60.1 ± 0.5	67.7 ± 0.5
400	20	47.9 ± 2.3	1.23 ± 0.00	58.7 ± 2.9	43.7 ± 1.9
	40	41.2 ± 0.4	1.34 ± 0.00	55.3 ± 0.6	58.0 ± 0.4
	60	38.3 ± 1.6	1.45 ± 0.00	55.6 ± 2.3	72.8 ± 2.0
450	20	42.8 ± 1.2	1.31 ± 0.00	55.9 ± 1.5	53.4 ± 1.1
	40	35.4 ± 0.6	1.42 ± 0.00	50.1 ± 0.8	64.4 ± 0.6
	60	34.2 ± 0.3	1.47 ± 0.00	50.3 ± 0.4	71.9 ± 0.3
500	20	36.5 ± 0.5	1.41 ± 0.00	51.5 ± 0.7	64.6 ± 0.5
	40	32.9 ± 0.4	1.48 ± 0.00	48.8 ± 0.5	71.9 ± 0.4
	60	32.0 ± 0.5	1.50 ± 0.00	48.0 ± 0.7	73.4 ± 0.5

**Table 4:** The fuel properties of biochar. The raw data can be found in the Supplementary Materials (sheet: ‘BC fuel properties’).

T, °C	t, min	VS, %	VM, %	FC, %	AC, %	HHV, J × g^−1^
Raw	Raw	97.2 ± 0.4	75.5 ± 0.5	21.7 ± 0.7	2.8 ± 0.4	18,312 ± 235
200	20	97.1 ± 0.2	75.0 ± 0.6	22.1 ± 0.7	2.9 ± 0.2	18,484 ± 413
	40	97.2 ± 0.4	72.8 ± 0.7	24.3 ± 0.8	2.8 ± 0.4	18,820 ± 247
	60	96.7 ± 0.2	69.6 ± 0.6	27.1 ± 0.8	3.3 ± 0.2	19,389 ± 450
250	20	97.1 ± 0.0	73.2 ± 1.6	23.9 ± 1.6	2.9 ± 0.0	18,704 ± 646
	40	96.0 ± 0.4	65.0 ± 0.6	31.0 ± 0.8	4.0 ± 0.4	21,463 ± 396
	60	96.2 ± 0.3	54.8 ± 0.5	41.4 ± 0.7	3.8 ± 0.3	21,633 ± 431
300	20	94.7 ± 0.5	64.5 ± 1.1	30.2 ± 1.3	5.3 ± 0.5	20,102 ± 377
	40	94.1 ± 0.5	45.9 ± 0.7	48.2 ± 0.9	5.9 ± 0.5	24,720 ± 267
	60	92.9 ± 0.3	40.6 ± 0.3	52.3 ± 0.1	7.1 ± 0.3	25,113 ± 731
350	20	89.8 ± 3.5	37.4 ± 0.9	52.3 ± 2.6	10.2 ± 3.5	23,096 ± 251
	40	92.0 ± 1.5	36.1 ± 1.4	55.9 ± 2.2	8.0 ± 1.5	24,862 ± 601
	60	91.7 ± 1.1	46.9 ± 0.8	44.8 ± 1.3	8.3 ± 1.1	25,324 ± 49
400	20	91.8 ± 1.8	43.9 ± 3.4	47.8 ± 1.6	8.2 ± 1.8	22,478 ± 811
	40	91.0 ± 2.7	34.7 ± 1.5	56.3 ± 1.9	9.0 ± 2.7	24,548 ± 594
	60	90.1 ± 1.6	40.0 ± 0.6	50.0 ± 1.0	9.9 ± 1.6	26,531 ± 251
450	20	90.7 ± 2.1	36.2 ± 1.7	54.5 ± 1.1	9.3 ± 2.1	23,903 ± 846
	40	88.5 ± 1.1	25.0 ± 2.0	63.5 ± 3.1	11.5 ± 1.1	25,926 ± 417
	60	89.3 ± 3.4	32.9 ± 1.0	56.4 ± 4.1	10.7 ± 3.4	26,979 ± 400
500	20	87.8 ± 1.9	30.0 ± 1.1	57.8 ± 2.9	12.2 ± 1.9	25,815 ± 348
	40	88.8 ± 0.5	26.5 ± 0.0	62.2 ± 0.5	11.2 ± 0.5	27,139 ± 452
	60	85.9 ± 2.1	29.0 ± 0.2	56.9 ± 1.9	14.1 ± 2.1	27,443 ± 168

**Table 5:** The results of pollutants in biochar water extracts.

T, °C	t, min	Total Carbon Content, ppm	Organic Carbon Content, ppm	Inorganic Carbon Content, ppm	Total Nitro-gen, mg N × dm^−3^	Kjeldahl Nitrogen, mg N × dm^−3^	Organic Nitrogen, mg N_org_ × dm^−3^	Ammonical Nitrogen, mg N_NH4_ × dm^−3^	Nitric Nitrogen, mg N_NO3_ × dm^−3^	Total Phosphorus, mg P × dm^−3^	Dissolved Oxygen, mg O_2_ × dm^−3^	BOD_5_, mg O_2_ × dm^−3^	COD (Cr), mg O_2_ × dm^−3^	General Suspensions, mg × dm^−3^	General Dis-solved Sub-stances, mg × dm^−3^	pH	Conductivity, μS × cm^−1^
Raw	Raw	4680 ± 18	4760 ± 25	80 ± 7	224.79	216.51	164.91	51.60	8.28	86.69	0.0	3048	6850	1865	4250	5.5	2530
200	20	2948 ± 36	3028 ± 27	81 ± 9	422.04	353.50	299.05	54.49	68.5	37.70	1.2	1320	12,074	1610	9210	5.8	2890
	40	2468 ± 2	2544 ± 4	78 ± 5	213.33	130.53	127.43	3.10	82.8	78.04	1.4	692	4674	20	6420	5.8	2080
	60	4840 ± 61	4920 ± 61	90 ± 0	154.49	108.19	93.91	14.28	46.3	90.94	1.0	812	2804	375	5595	6.0	2480
250	20	4400 ± 25	4520 ± 25	88 ± 0	330.40	265.60	255.2	10.4	64.8	80.61	1.6	548	8335	795	5870	5.7	1240
	40	2896 ± 38	2980 ± 39	84 ± 1	315.33	239.93	234.33	5.60	75.4	135.8	1.2	604	7946	360	9105	5.6	2770
	60	4360 ± 129	4440 ± 140	91 ± 10	240.14	139.04	129.66	9.38	101.1	93.94	1.4	722	3895	470	6530	5.8	1587
300	20	1756 ± 100	1912 ± 78	153 ± 22	263.53	190.96	180.08	10.88	72.57	49.62	1.8	281	7011	240	9235	5.7	1351
	40	1625 ± 98	1760 ± 98	135 ± 1	87.20	77.85	75.62	2.23	0.56	93.26	3.8	1672	7646	212	5694	5.8	3310
	60	700 ± 37	752 ± 42	56 ± 5	69.71	67.47	65.99	1.48	0.38	81.1	3.0	1866	5470	47	5576	6.3	3100
350	20	520 ± 19	572 ± 14	51 ± 5	113.90	105.51	103.49	2.02	8.39	97.62	4.2	1408	5764	735	5955	5.9	2330
	40	2332 ± 37	2380 ± 35	48 ± 2	113.20	33.91	33.25	0.66	79.29	45.99	4.6	108	1664	160	2430	7.6	1838
	60	266 ± 10	324 ± 10	58 ± 0	32.80	24.43	24.07	0.36	8.38	23.49	4.4	400	1149	140	1725	7.7	1533
400	20	139 ± 0	212 ± 0	72 ± 0	149.55	87.05	86.26	0.79	62.5	9.63	4.4	1056	6070	170	6210	5.8	2380
	40	50 ± 3	94 ± 2	44 ± 2	18.02	10.65	10.57	0.08	7.37	15.55	3.6	158	579	100	1230	8.6	1163
	60	892 ± 16	1040 ± 10	149 ± 6	11.74	5.12	5.10	0.02	6.62	4.60	5.0	132	231	50	955	7.5	1127
450	20	190 ± 7	373 ± 1	183 ± 8	58.75	41.30	40.76	0.54	0.2	33.42	4.6	1034	2112	62	2139	7.0	1636
	40	134 ± 0	278 ± 0	143 ± 1	16.71	15.98	15.83	0.15	nf	9.52	6.0	275	594	705	830	9.7	1368
	60	230 ± 4	352 ± 5	122 ± 10	12.49	12.27	12.11	0.16	0.22	13.71	6.0	196	2373	560	733	10.8	2040
500	20	200 ± 10	346 ± 9	146 ± 1	17.52	17.31	17.21	0.10	0.21	13.88	6.2	202	439	134	517	8.4	796
	40	158 ± 13	323 ± 3	164 ± 10	18.26	14.81	14.69	0.12	0.15	13.52	6.0	63	102	1046	1051	10.6	2050
	60	4680 ± 18	4760 ± 25	80 ± 7	87.20	13.63	75.62	0.13	0.15	12.42	5.8	45	375	107	1170	10.8	2190

nf—not found.

**Table 6:** Metal contents of biochar extracts.

T, °C	t, min	Sodium, mg Na × dm^−3^	Potassium, mg K ×·dm^−3^	Calcium, mg Ca ×·dm^−3^	Magnesium, mg Mg ×·dm^−3^	Zinc, μg Zn ×·dm^−3^	Copper, μg Cu ×·dm^−3^	Cadmium, μg Cd ×·dm^−3^	Lead, μg Pb ×·dm^−3^	Chromium, μg Cr ×·dm^−3^	Nickel, μg Ni ×·dm^−3^	Manganese, mg Mn ×·dm^−3^	Iron, mg Fe ×·dm^−3^
Raw	Raw	21.6	504.4	71.7	58.6	1464.0	312.5	nf	nf	133.0	31.6	2.7	1.15
200	20	31.0	675.0	173.2	85.9	2335.8	1311.0	13.5	nf	389.0	156.5	5.86	2.36
	40	29.5	685.1	152.8	73.2	1829.5	1535.0	1.5	nf	187.5	570.0	5.0	2.53
	60	29.1	650.8	143.1	64.0	1547.5	646.5	10.5	nf	268.5	20.0	3.88	2.1
250	20	31.0	676.4	158.0	84.4	2442.5	318.0	nf	nf	250.0	nf	6.42	3.19
	40	26.3	550.5	145.8	74.3	2104.5	222.5	5.0	nf	145.0	nf	6.07	0.93
	60	30.1	693.5	164.1	81.0	1834.0	218.0	nf	nf	366.0	105.0	5.4	4.6
300	20	32.0	677.9	178.1	92.9	2113.5	299.0	1.5	nf	534.0	143.0	7.44	1.7
	40	28.9	331.3	186.9	57.2	1999.5	76.3	1.6	nf	83.2	85.8	7.7	0.21
	60	25.3	338.3	172.6	49.3	474.6	56.5	1.4	nf	80.4	73.9	4.07	0.13
350	20	22.2	578.9	77.8	65.6	1706.5	300.0	nf	nf	125.0	22.4	2.94	0.55
	40	18.4	496.2	40.8	27.7	852.5	234.5	nf	nf	103.5	16.0	0.6	0.63
	60	17.5	392.6	42.2	29.0	784.0	214.5	nf	nf	85.5	18.0	1.02	0.53
400	20	24.5	562.0	117.8	72.0	1354.5	288.0	nf	nf	75.5	23.9	4.87	0.87
	40	14.7	328.3	19.4	15.7	757.5	202.0	nf	nf	66.0	nf	0.27	0.38
	60	11.5	305.9	12.5	9.7	727.0	212.5	nf	nf	105.5	nf	0.12	12.15
450	20	14.6	305.6	59.2	24.5	135.6	52.1	nf	nf	65.7	51.3	0.77	0.04
	40	10.5	284.8	10.6	7.8	125.9	33.3	nf	nf	52.7	40.7	0.06	2.92
	60	13.2	314.5	11.5	6.1	118.8	38.3	nf	nf	57.6	37.0	0.02	0.29
500	20	7.0	232.4	9.9	10.2	120.3	35.2	0.7	nf	71.7	62.3	0.14	0.27
	40	10.5	302.9	11.2	7.5	102.3	36.7	0.9	nf	55.4	32.4	0.03	0.13
	60	12.7	315.2	8.9	5.6	97.1	39.4	nf	nf	61.5	35.0	0.01	0.08

nf—not found.

**Table 7:** Phytotoxicity results of biochar. The raw data can be found in the Supplementary Materials (sheet: ‘Root length and germination’).

T, °C	t, min	Average Root Length, mm	Inhibition of Root Elongation, %	Average Seed Germination, %
200	20	17.08 ± 3.56	68.83 ± 3.56	13.33 ± 3.56
	40	21.92 ± 6.06	60.00 ± 6.06	53.33 ± 6.06
	60	41.61 ± 14.46	24.07 ± 14.46	73.33 ± 14.46
250	20	26.19 ± 11.50	52.21 ± 11.50	36.67 ± 11.50
	40	41.58 ± 19.21	24.12 ± 19.21	83.33 ± 19.21
	60	55.67 ± 15.84	−1.59 ± 15.84	80.00 ± 15.84
300	20	36.53 ± 18.48	33.34 ± 18.48	86.67 ± 18.48
	40	51.8 ± 14.96	5.47 ± 14.96	96.67 ± 14.96
	60	52.35 ± 23.77	4.47 ± 23.77	90.00 ± 23.77
350	20	44.85 ± 21.33	18.15 ± 21.33	90.00 ± 21.33
	40	44.75 ± 19.25	18.34 ± 19.25	96.67 ± 19.25
	60	54.02 ± 17.38	1.42 ± 17.38	90.00 ± 17.38
400	20	66.98 ± 7.35	−22.23 ± 7.35	93.33 ± 7.35
	40	64.16 ± 16.79	−17.09 ± 16.79	86.67 ± 16.79
	60	57.38 ± 21.09	−4.71 ± 21.09	93.33 ± 21.09
450	20	59.61 ± 20.48	−8.78 ± 20.48	96.67 ± 20.48
	40	72.16 ± 14.67	−31.68 ± 14.67	96.67 ± 14.67
	60	52.56 ± 23.34	4.08 ± 23.34	90.00 ± 23.34
500	20	57.95 ± 24.47	−5.75 ± 24.47	86.67 ± 24.47
	40	64.05 ± 24.14	−16.88 ± 24.14	100.00 ± 24.14
	60	56.69 ± 20.12	−3.45 ± 20.12	90.00 ± 20.12

## Data Availability

All measured and generated data on biochar production and proximate analysis, as well as biochar phytoxicity, can be found in the Supplementary Materials.

## Supplementary Materials

The following supporting information can be downloaded at: https://www.mdpi.com/article/10.3390/ma17081895/s1, The Excel Spreadsheet file “Supplementary Material” contains the following sheets with raw data: ‘BC production’, ‘BC fuel properties’, and ‘Roots length and germination’.
